# Interferon-γ elicits the ocular surface pathology mimicking dry eye through direct modulation of resident corneal cells

**DOI:** 10.1038/s41420-023-01511-0

**Published:** 2023-06-30

**Authors:** Jung Hwa Ko, Seonghwan Kim, Jin Suk Ryu, Hyo Jeong Song, Joo Youn Oh

**Affiliations:** 1 Laboratory of Ocular Regenerative Medicine and Immunology, Biomedical Research Institute, Seoul National University Hospital, 101 Daehak-ro, Jongno-gu, Seoul, 03080 Korea; 2 Department of Ophthalmology, Seoul Metropolitan Government Seoul National University Boramae Medical Center, 20 Boramae-ro 5 Gil, Dongjak-gu, Seoul, 07061 Korea; 3 Department of Ophthalmology, Seoul National University College of Medicine, 103 Daehak-ro, Jongno-gu, Seoul, 03080 Korea

**Keywords:** Corneal diseases, Experimental models of disease

## Abstract

Despite accumulating evidence indicating a key role of interferon-γ (IFN-γ)-producing immune cells in ocular infection and immunity, little is known about the direct effects of IFN-γ on resident corneal cells or on the ocular surface. Here, we report that IFN-γ impacts corneal stromal fibroblasts and epithelial cells to promote inflammation, opacification, and barrier disruption on the ocular surface, leading to dry eye. Our results demonstrated that IFN-γ dose-dependently induced cytotoxicity, pro-inflammatory cytokine/chemokine production, and expression of major histocompatibility complex class II and CD40 in cultures of corneal stromal fibroblasts and epithelial cells while increasing myofibroblast differentiation of corneal stromal fibroblasts. In mice, subconjunctival IFN-γ administration caused corneal epithelial defects and stromal opacity in dose- and time-dependent manners while promoting neutrophil infiltration and inflammatory cytokine expression in the cornea. Moreover, IFN-γ reduced aqueous tear secretion and the number of conjunctival goblet cells responsible for mucinous tear production. Together, our findings suggest that IFN-γ induces the ocular surface changes characteristic of dry eye disease at least in part through its direct effects on resident corneal cells.

## Introduction

Interferon-γ (IFN-γ), the only type II interferon, is a pleiotropic cytokine that regulates both innate and adaptive immune responses in infectious and immune-mediated diseases [1–3]. Multiple data from animal models and human patients implicate IFN-γ in the induction and protection of various ocular diseases ranging from infection to transplant rejection to dry eye disease (DED). For example, IFN-γ-producing invariant natural killer T (iNKT) cells alleviated the symptoms of ocular herpesvirus infection in mice [4], while IFN-γ gene knockout (KO) mice exhibited higher disease severity or virus reactivation rates after herpes simplex virus (HSV) type 1 infection as compared with wild-type mice [5, 6], which results suggest a protective role of IFN-γ in ocular infection. On the other hand, IFN-γ-expressing Th17 cells or natural killer (NK) cells initiated an immune response or aggravated corneal epitheliopathy in desiccating stress-induced DED mice [7, 8], while IFN-γ deletion prevented dacryoadenitis in CD25 KO DED mice [9, 10]. Moreover, IFN-γ levels in tear fluid or conjunctiva were increased in patients with DED, ocular graft–versus–host disease (GVHD), and vernal or atopic keratoconjunctivitis [11–16]. These findings indicate a pathogenic role of IFN-γ in non-infectious ocular immune diseases. Meanwhile, studies with corneal transplant models have produced more complex results and shown paradoxical effects of IFN-γ, depending on the disease context. Elevated IFN-γ levels were demonstrated in the aqueous humor of patients undergoing immune rejection of corneal grafts [17, 18], pointing to an immunogenic role of IFN-γ in transplantation. By contrast, IFN-γ deficiency in IFN-γ KO or anti-IFN-γ-treated mice led to higher rejection rates of fully allogeneic (both major histocompatibility complex (MHC)- and minor histocompatibility (minor H)-mismatched) corneal grafts, indicating the contribution of IFN-γ to immune privilege, but conversely promoted survival of either MHC-mismatched (and minor H-matched) grafts or minor H-mismatched (and MHC-matched) grafts [19].

These context-dependent, paradoxical effects of IFN-γ have also been observed at cellular levels in studies using fibroblasts from different tissues. IFN-γ-secreting Th1 cells were shown to induce the transition of cardiac fibroblasts into myofibroblasts and thus drive cardiac fibrosis [20]. Conversely, it was reported that IFN-γ downregulated the differentiation of lung and skin fibroblasts to myofibroblasts [21–25], demonstrating the potential of IFN-γ therapy in patients with idiopathic pulmonary fibrosis or keloids.

Despite accumulating data on the pathogenic or therapeutic role of IFN-γ according to disease and tissue contexts, little is known about its direct impacts on resident corneal cells or on the ocular surface. Thus, in the present study, we investigated the dose-dependent effects of IFN-γ on the survival, inflammatory activation and differentiation of corneal stromal fibroblasts and epithelial cells in vitro, and compared them with those of interleukin (IL)-17, the canonical cytokine of Th17 cells that are implicated in the pathogenesis of various ocular surface disorders alongside IFN-γ-producing cells. Further, we assessed the time- and dose-dependent effects of subconjunctival IFN-γ administration on the ocular surface including the cornea, conjunctiva, and lacrimal glands in mice.

## Results

### IFN-γ, but not IL-17, reduces the viability of corneal stromal fibroblasts and epithelial cells

To evaluate the direct effects of IFN-γ and IL-17 on cell viability, we treated primary cultures of human corneal stromal fibroblasts and epithelial cells with various concentrations of IFN-γ or IL-17 (10–100 ng/mL) for 18 h, and assessed the cells’ metabolic activity and cytotoxicity.

**Fig. 1:**
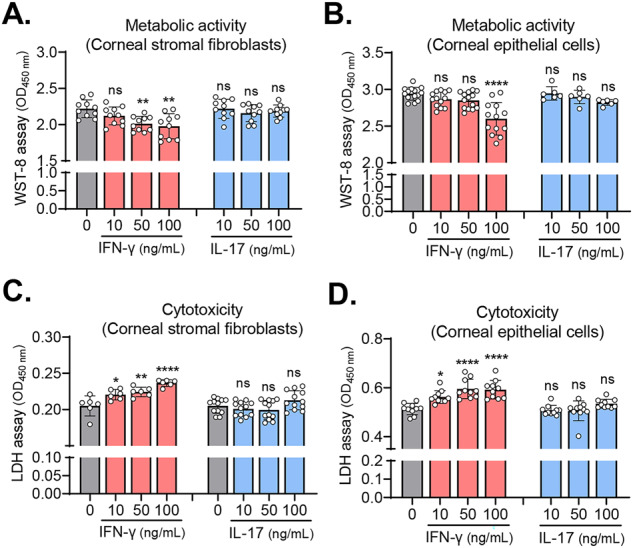
IFN-γ reduces cell viability in corneal stromal fibroblasts and epithelial cells, whereas IL-17 does not. **A**, **B** Quantification of metabolic activity of human corneal stromal fibroblasts (**A**) and corneal epithelial cells (**B**) treated with either IFN-γ or IL-17, as assessed by WST-8 assay. **C, D**. Quantification of LDH activity in culture supernatants of human corneal stromal fibroblasts (**C**) and corneal epithelial cells (**D**) treated with either IFN-γ or IL-17. Mean values ± SD are shown. Asterisks indicate the values relative to the control group (cells not treated with either IFN-γ or IL-17). *P < 0.05, **P < 0.01, ****P < 0.0001, ns: not significant, as analyzed by one-way ANOVA with Tukey’s multiple-comparison test.

The WST-8 assay revealed that IFN-γ dose-dependently decreased the metabolic activity in corneal stromal fibroblasts and epithelial cells (Fig. 1A, B). Similarly, quantification of lactate dehydrogenase (LDH) release demonstrated that IFN-γ induced cytotoxicity in both cell types in a dose-dependent manner (Fig. 1C, D). IL-17, however, did not affect the metabolic activity or cause toxicity in either corneal stromal fibroblasts or corneal epithelial cells (Fig. 1A–D).

These results indicate that IFN-γ directly reduces cell viability in both corneal stromal fibroblasts and corneal epithelial cells, whereas IL-17 has no effects on cell survival.

### IFN-γ and, to a lesser degree, IL-17, increase inflammatory cytokines in corneal stromal fibroblasts and epithelial cells

We next investigated the effects of IFN-γ and IL-17 on the production of inflammatory cytokines and chemokines in corneal stromal fibroblasts and epithelial cells.

**Fig. 2:**
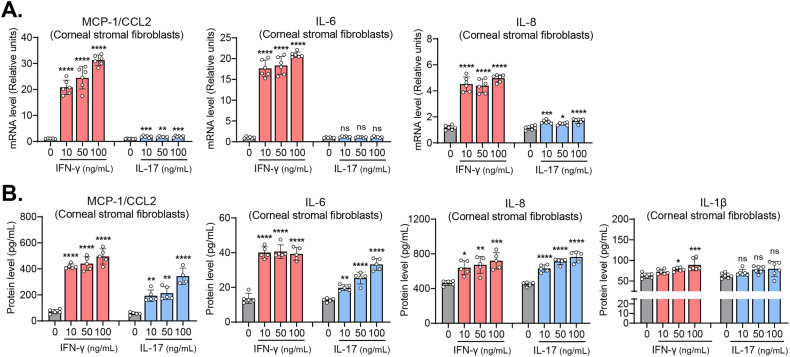
IFN-γ and IL-17 upregulate inflammatory cytokines and chemokines in corneal stromal fibroblasts. **A** RT-qPCR for inflammatory chemokines and cytokines (MCP-1/CCL2, IL-6, IL-8) in human corneal stromal fibroblasts treated with incremental doses of IFN-γ or IL-17. The mRNA levels are shown as fold changes relative to the control group that was not treated with IFN-γ or IL-17. **B** ELISA for secreted levels of MCP-1/CCL2, IL-6, IL-8, and IL-1β in human corneal stromal fibroblasts treated with IFN-γ or IL-17. Mean values ± SD are shown. Asterisks indicate the values relative to the control group. *P < 0.05, **P < 0.01, ***P < 0.001, ****P < 0.0001, ns not significant, as analyzed by one-way ANOVA with Tukey’s multiple-comparison test.

Our results showed that IFN-γ markedly increased the transcript levels and the secreted levels of MCP-1/CCL2, IL-6, IL-8, and IL-1β in corneal stromal fibroblasts (Fig. 2A, B). IL-17 also upregulated the transcript or the secreted levels of MCP-1/CCL2, IL-6, and IL-8 in corneal stromal fibroblasts. However, the effects of IL-17 on the transcription or secretion of MCP-1/CCL2, IL-6, and IL-8 were smaller relative to those of IFN-γ. Moreover, IL-17 did not significantly affect IL-1β secretion in corneal fibroblasts (Fig. 2B).

**Fig. 3:**
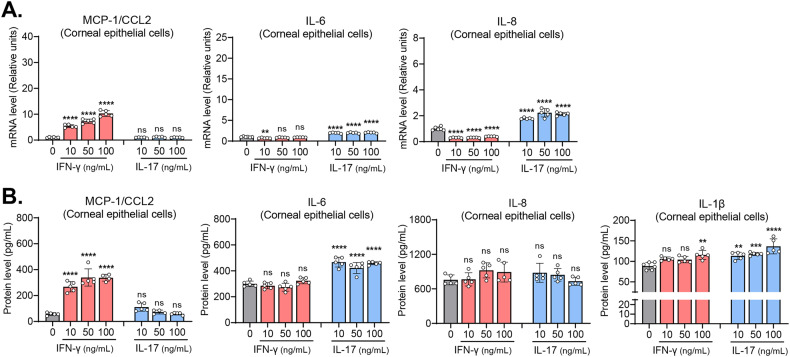
Effects of IFN-γ and IL-17 on the expression of inflammatory cytokines and chemokines in corneal epithelial cells. **A** RT-qPCR for inflammatory chemokines and cytokines (MCP-1/CCL2, IL-6, IL-8) in human corneal epithelial cells treated with IFN-γ or IL-17. The mRNA levels are presented as fold changes relative to the control group not treated with IFN-γ or IL-17. **B**. ELISA for secreted levels of MCP-1/CCL2, IL-6, IL-8, and IL-1β in human corneal epithelial cells treated with IFN-γ or IL-17. Mean values ± SD are shown. Asterisks indicate the values relative to the control group. **P < 0.01, ***P < 0.001, ****P < 0.0001, ns: not significant, as analyzed by one-way ANOVA with Tukey’s multiple-comparison test.

In parallel experiments with corneal epithelial cells, IFN-γ increased both transcript and secreted levels of MCP-1/CCL2 in a dose-dependent manner, whereas IL-17 did not impact MCP-1/CCL2 in the cells (Fig. 3A, B). By contrast, IL-17 significantly increased the transcript or secreted levels of IL-6, IL-8, and IL-1β in corneal epithelial cells, whereas IFN-γ did not increase IL-6 or IL-8 in the cells (Fig. 3A, B).

Together, the data demonstrate that IFN-γ is more potent than IL-17 in the induction of inflammatory cytokines and chemokines in corneal stromal fibroblasts, whereas IL-17 is more effective at inflammatory cytokine induction in corneal epithelial cells. Notably, corneal stromal fibroblasts produce higher levels of chemokines and inflammatory cytokines, such as MCP-1/CCL2, IL-6, IL-8, and IL-1β, in response to IFN-γ stimulation than do corneal epithelial cells. Therefore, the results thus far obtained indicate that corneal stromal fibroblasts might be one of the major cell populations that amplify the pro-inflammatory signals upon injury and recruit immune cells to the cornea, and that IFN-γ drives activation of corneal stromal fibroblasts.

### IFN-γ, but not IL-17, induces MHC class II and CD40 on corneal stromal fibroblasts and epithelial cells

To further analyze immunogenicity, we examined the surface expression of MHC class II and co-stimulatory molecules CD40, CD80, and CD86 on corneal stromal fibroblasts and epithelial cells before and after treatment with IFN-γ or IL-17.

**Fig. 4:**
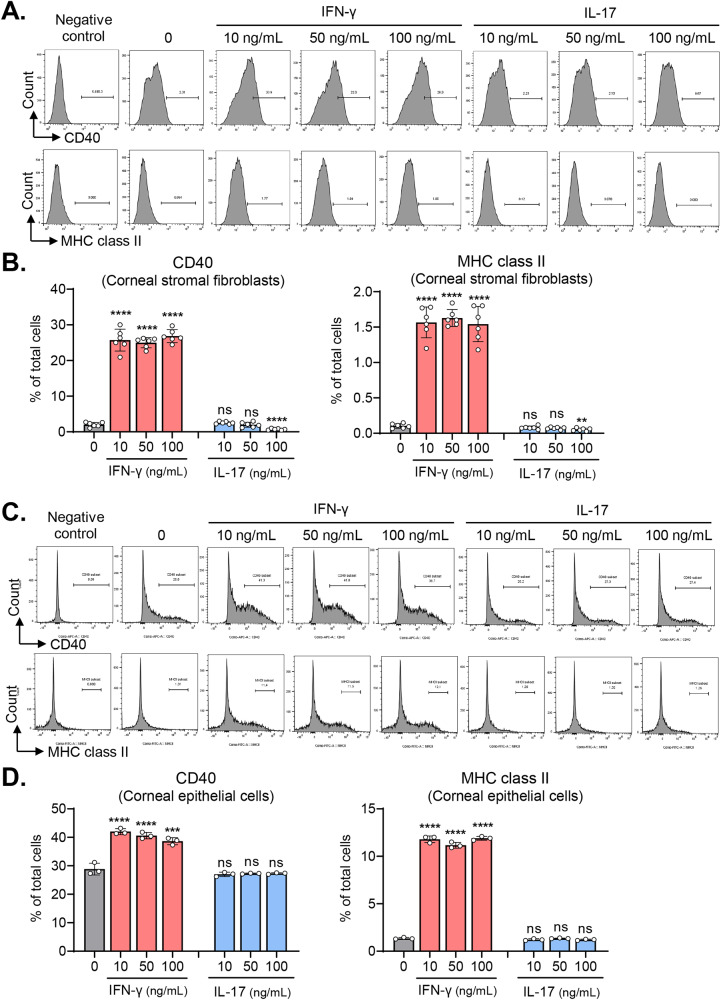
IFN-γ induces MHC class II and CD40 expression on corneal stromal fibroblasts and corneal epithelial cells, whereas IL-17 does not. **A**, **B** Representative flow cytometry histograms and quantification of CD40 and MHC class II in human corneal stromal fibroblasts treated with IFN-γ or IL-17. **C**, **D** Representative and quantitative flow cytometry results for CD40 and MHC class II in human corneal epithelial cells treated with IFN-γ or IL-17. The data are presented in mean values ± SD. Asterisks indicate the values relative to cells not treated with IFN-γ or IL-17. **P < 0.01, ***P < 0.001, ****P < 0.0001, ns not significant, by one-way ANOVA with Tukey’s multiple-comparison test.

The expression levels of CD40, MHC class II, CD80, and CD86, as measured by flow cytometry, were negligible in corneal stromal fibroblasts without stimulation (Fig. 4A, B and Supplementary Fig. S1). Stimulation of the cells with IFN-γ (10–100 ng/mL) induced the expression of CD40 and MHC class II on the surface of corneal stromal fibroblasts (Fig. 4A, B), while the levels of CD80 and CD86 were unaltered (Supplementary Fig. S1). Conversely, IL-17 suppressed the expression of CD40 and MHC class II on corneal stromal fibroblasts at a higher concentration (100 ng/mL) (Fig. 4B). Similar results were obtained with corneal epithelial cells. IFN-γ stimulation significantly enhanced the expression of CD40 and MHC class II on corneal epithelial cells (Fig. 4C, D). One notable finding was that corneal epithelial cells expressed higher levels of CD40 and MHC class II at steady state, as compared to corneal stromal fibroblasts (Fig. 4A–D), which is consistent with the observations made with epithelial cells of the gastrointestinal and respiratory tracts [26].

These findings suggest that IFN-γ might render resident corneal cells immunogenic, thereby contributing to the disruption of ocular immune privilege.

### IFN-γ, but not IL-17, promotes myofibroblast differentiation of corneal stromal fibroblasts

IFN-γ and IL-17 have been implicated in the development of fibrosis in the heart, liver, and intestines through triggering myofibroblast differentiation [20, 27, 28]. Contrastingly, IFN-γ has also been shown to suppress the myofibroblast transition of skin and lung fibroblasts and inhibit fibrosis in the skin and lung [22, 23]. These findings demonstrate both pro- and anti-fibrotic roles of IFN-γ according to the tissue context. Thus, we next investigated the influence of IFN-γ and IL-17 on myofibroblast differentiation of corneal stromal fibroblasts.

**Fig. 5:**
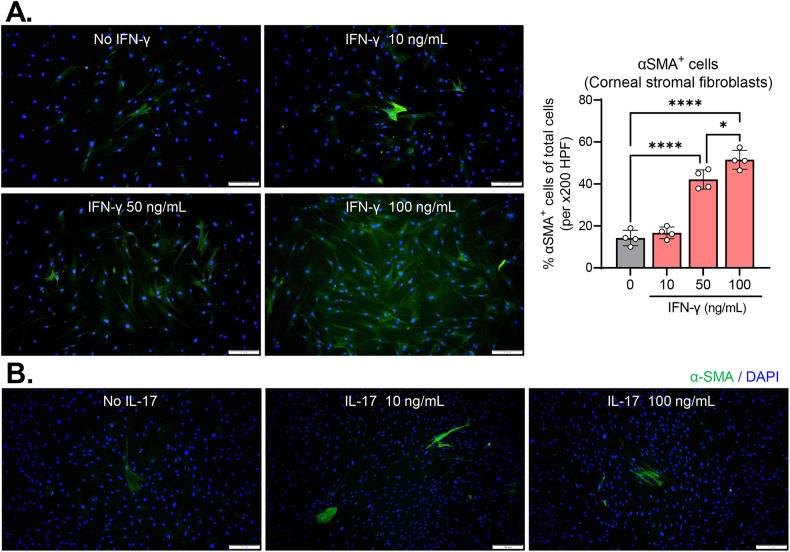
IFN-γ dose-dependently induces myofibroblast differentiation in corneal stromal fibroblasts, whereas IL-17 does not. Representative microphotographs of corneal stromal fibroblasts stained with α-SMA after treatment with IFN-γ (**A**) or IL-17 (**B**). Scale bar: 200 μm. The quantification of α-SMA^+^ cells in IFN-γ-treated corneal stromal fibroblasts was expressed as the percentage of α-SMA^+^ cells relative to the total number of cells. *P < 0.05, ****P < 0.0001, as analyzed by one-way ANOVA with Tukey’s multiple-comparison test.

Immunostaining for α-smooth muscle actin (α-SMA), a hallmark of fibroblast differentiation to myofibroblasts, revealed that IFN-γ increased the number of α-SMA-expressing cells in cultures of corneal stromal fibroblasts in a dose-dependent manner (Fig. 5A). IL-17, however, did not exhibit any such effects (Fig. 5B).

In the aggregate, our results suggest that IFN-γ has potential pro-fibrotic effects on the cornea, both directly through induction of myofibroblast differentiation in corneal stromal fibroblasts and indirectly through promotion of inflammation.

### Subconjunctival IFN-γ induces corneal stromal opacity, epithelial defects, and inflammation

Having observed that IFN-γ reduced cell survival, induced inflammatory activation, and elicited myofibroblast transition in resident corneal cells in vitro, we determined to confirm whether these effects of IFN-γ are reproduced in vivo.

**Fig. 6:**
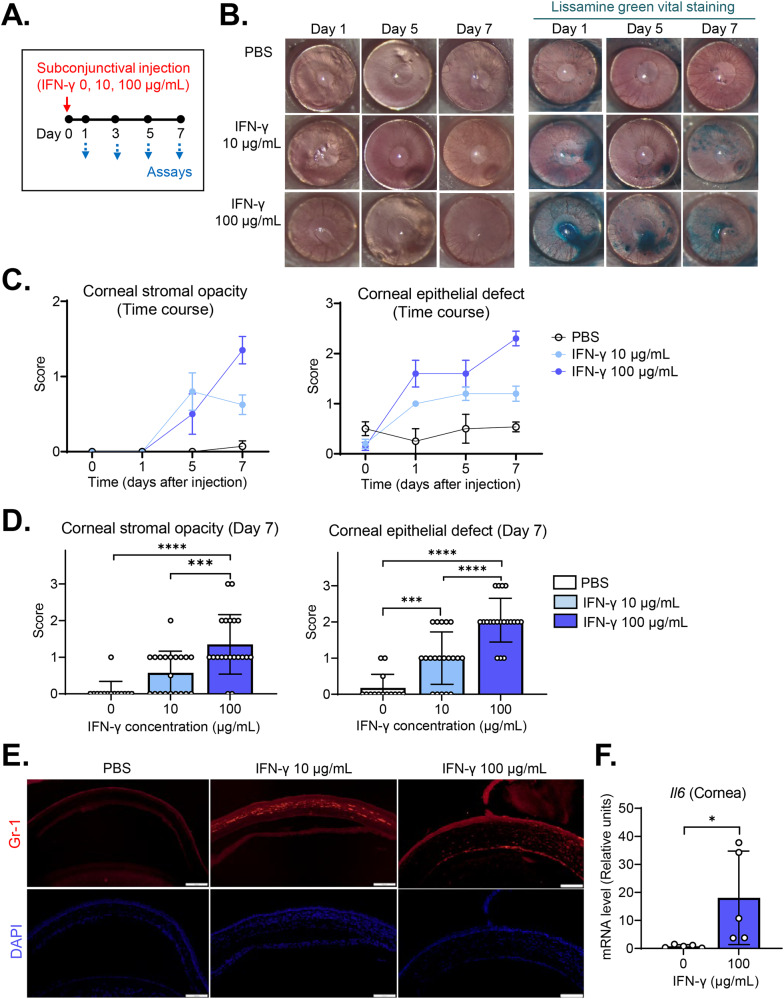
Subconjunctival IFN-γ administration induces corneal stromal opacity, punctate epithelial erosions, and inflammation on the ocular surface in mice. **A** Experimental scheme. Eight-week-old BALB/c mice were given IFN-γ (10 or 100 μg/mL in 5 μL PBS) via subconjunctival injection on day 0, after which they were followed up for clinical examination every other day and sacrificed for molecular and histological assays on day 7. **B** Representative corneal photographs before and after lissamine green vital dye staining on days 1, 5, and 7 following subconjunctival IFN-γ injection. The green-stained area reflects parts of the cornea with epithelial damage. **C** Time course of corneal stromal opacity and epithelial defects after subconjunctival IFN-γ treatment, as graded by standardized scoring systems. **D** Quantification of corneal stromal opacity and epithelial defects on day 7 after subconjunctival IFN-γ injection (*n* = 14 eyes for the control group; *n* = 20 eyes for IFN-γ 10 μg/mL group; *n* = 20 for IFN-γ 100 μg/mL group). **E** Representative microphotographs of corneal sections with Gr-1 immunostaining on day 7 after subconjunctival IFN-γ injection. Magnification ×200. Scale bar: 100 μm. **F** mRNA levels of IL-6 in the cornea as analyzed by RT-qPCR. The values are shown relative to those in control eyes without IFN-γ treatment. The data are presented in mean values ± SEM (**C**) or SD (**D**, **F**), where each circle indicates data from an individual mouse. *P < 0.05, ***P < 0.001, ****P < 0.0001, as analyzed by one-way ANOVA and Tukey’s test (**D**) or Student’s *t* test (**F**).

We injected 10 or 100 μg/mL recombinant IFN-γ in 5 μL of phosphate-buffered saline (PBS) or the same volume of PBS into the subconjunctival space of the eyes in BALB/c mice (day 0), and observed clinically for corneal stromal transparency and epithelial integrity every other day for 7 days (Fig. 6A). At day 7, the aqueous tear volume was measured, and tissues including the cornea, conjunctiva, and lacrimal glands were extracted for molecular and histologic assays.

Slit-lamp biomicroscopic examination showed that the corneas of the IFN-γ-treated eyes had developed stromal opacity by day 5, and that the opacity was aggravated thereafter (Fig. 6B, C). Punctate epithelial erosions in the cornea, as measured with lissamine green vital dye staining, appeared earlier (being apparent at day 1 after IFN-γ injection) and gradually worsened until day 7 (Fig. 6B, C). Both corneal stromal opacity and epithelial defects at day 7 were significantly higher in 10 μg/mL or 100 μg/mL IFN-γ-treated eyes as compared with PBS-treated control eyes (Fig. 6D), and the effects of IFN-γ were dose-dependent: 100 μg/mL IFN-γ was significantly more potent than 10 μg/mL IFN-γ at the induction of corneal stromal opacity and epithelial erosions (Fig. 6D).

Immunostaining of corneal sections demonstrated marked infiltration of neutrophils (Gr-1^+^ cells) into the cornea of IFN-γ-treated eyes, whereas there were no neutrophils in PBS-treated controls (Fig. 6E). A similar pattern was observed in the data obtained by real-time reverse transcription-quantitative polymerase chain reaction (RT-qPCR) of the cornea. The mRNA levels of pro-inflammatory cytokine IL-6 were significantly increased in IFN-γ-treated eyes as compared with PBS-treated controls (Fig. 6F).

### Subconjunctival IFN-γ reduces tear production and conjunctival goblet cell (GC) counts

Punctate epithelial erosions and inflammation in the cornea are prominent features of DED. Since IFN-γ led to DED-like changes in the cornea (Fig. 6), we went on to explore the effects of IFN-γ on lacrimal glands and conjunctival GCs, which produce the aqueous and mucin layers of the tear film, respectively [29].

**Fig. 7:**
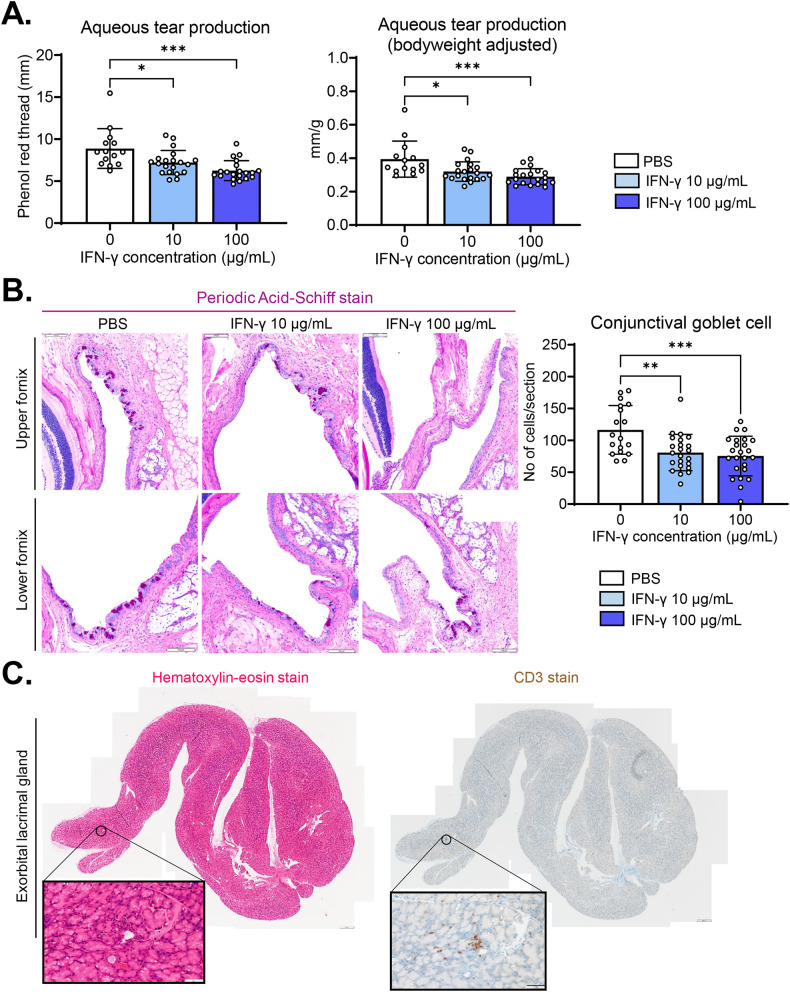
Subconjunctival IFN-γ administration reduces tear production and conjunctival GCs while causing focal T-cell infiltration into the lacrimal gland. **A** Quantification of aqueous tear production using phenol red thread test on day 7 after subconjunctival IFN-γ injection. **B** Representative microphotographs of upper and lower conjunctival fornices with PAS staining and quantification of mucin-secreting conjunctival GCs as a number of PAS-stained cells per eye. Magnification ×200. Scale bar: 100 μm. **C** Hematoxylin and eosin staining and CD3 immunostaining of exorbital lacrimal gland, the main glands for aqueous tear production in mice, on day 7 after subconjunctival IFN-γ injection (100 μg/mL). Magnification ×100 (main) and x400 (inset). Scale bars: 200 μm (main) and 50 μm (inset). Mean values ± SD are shown. Each circle indicates the data from an individual mouse. *P < 0.05, **P < 0.01, ***P < 0.001, ****P < 0.0001, ns: not significant, as analyzed by one-way ANOVA with Tukey’s multiple-comparison test.

Aqueous tear production, as quantified by phenol red thread test, was significantly impaired by subconjunctival IFN-γ administration (Fig. 7A). Likewise, Periodic Acid-Schiff (PAS) staining showed that the numbers of mucin-secreting conjunctival GCs were significantly lower in IFN-γ-treated eyes than in PBS-treated eyes (Fig. 7B). Moreover, CD3 immunostaining demonstrated focal infiltration of T cells into the exorbital lacrimal gland, the major murine gland responsible for aqueous tear production, in IFN-γ-treated eyes (Fig. 7C): there was focal CD3^+^ cell infiltration in one of five 10 μg/mL IFN-γ-treated glands and in three of five 100 μg/mL IFN-γ-treated glands, whereas there were no CD3^+^ cells in any of PBS-treated glands. CD3 cell infiltration was not observed in the intraorbital lacrimal gland or Harderian gland in IFN-γ-treated eyes or in PBS-treated controls (Supplementary Fig. S2).

Together, the data demonstrate that subconjunctival IFN-γ administration dose-dependently induces corneal stromal opacification and epithelial defects, reduces tear secretion and conjunctival GC numbers, and promotes inflammation in the cornea and lacrimal glands.

## Discussion

In this study, we determined that IFN-γ induced corneal stromal opacity, punctate epithelial erosions, corneal inflammation, lacrimal tear reduction, and conjunctival GC deficiency, all of which are characteristic of DED. The detrimental effects of IFN-γ on the ocular surface were explained by its actions on resident corneal cells: corneal stromal fibroblasts and epithelial cells were responsive to IFN-γ by undergoing cell death, immunogenic activation, and myofibroblast differentiation. IL-17, relative to IFN-γ, had less significant effects on resident corneal cells.

IFN-γ is the only member of type II interferon produced primarily by T lymphocytes and NK cells. According to previous reports, IFN-γ and IFN-γ-producing cells are implicated in the pathogenesis of a range of ocular surface diseases including DED [7, 8, 13], ocular Sjögren’s syndrome [3, 9, 10], ocular GVHD [12, 14], corneal allograft rejection [17, 18, 30, 31], and infection with HSV or pseudomonas [32–34]. Whereas IFN-γ exhibits both deleterious and protective effects on corneal graft rejection and infection, multiple studies have shown results consistent with the pathogenic role of IFN-γ in DED development and progression [3, 7–14). For instance, IFN-γ has been associated with loss of conjunctival GCs and lacrimal gland acinar cells in DED. Desiccating stress was found to induce migration of IFN-γ^+^ cells into the GC-rich zone of the conjunctiva and to decrease GC density [35]. Topical IFN-γ neutralization prevented conjunctival GC loss in a desiccating stress-induced dry eye model [36]. Similarly, IFN-γ deficiency led to lower numbers of apoptotic cells in the lacrimal gland and delayed onset of dacryoadenitis in the non-obese diabetic mouse and CD25 KO mouse models of Sjögren’s syndrome [9, 10, 37]. Also, IFN-γ treatment inhibited mucin secretion in cultures of GCs while reducing GC proliferation [38, 39]. NK cells and Th17 cells have been identified as the cellular sources that produce IFN-γ at the early and late stages of DED, respectively [7, 8].

In addition to these findings, our study found that IFN-γ directly induced cell death, upregulated secretion of inflammatory cytokines and chemokines, and increased expression of MHC class II and CD40 on corneal stromal fibroblasts and epithelial cells. Because dying cells act as DAMPs (damage-associated molecular patterns) that initiate immune responses, IFN-γ-induced cell death of resident corneal cells as well as their immunogenic activation would trigger and amplify the vicious cycle of ocular surface inflammation. Indeed, we observed that exogenous IFN-γ administration elicited a DED-like phenotype characterized by ocular surface barrier disruption and inflammation in naïve mice.

Another notable finding of our study is that, among the resident corneal cells, corneal stromal fibroblasts responded more dramatically to IFN-γ compared with corneal epithelial cells. Corneal stromal fibroblasts, also called keratocytes, are the principal cells of the corneal stroma that makes up about 90% of corneal thickness [40]. They are quiescent cells in the healthy cornea, playing a role in maintaining corneal transparency. Immediately after injury, however, keratocytes located at the injury site commit apoptosis, and neighboring keratocytes become activated as sentinel cells that sense external stimuli, recruit immune cells, and promote inflammation. Over a disease course, keratocytes are transformed into myofibroblasts expressing α-SMA, giving rise to corneal stromal fibrosis or opacification. In our study, IFN-γ activated corneal stromal fibroblasts to produce higher levels of pro-inflammatory cytokines and chemokines, upregulate surface expression of MHC class II and CD40, and trigger myofibroblast transformation. Consequently, IFN-γ administration into the eye of naïve mice led to corneal stromal opacification and inflammation. Given that excessive inflammatory reactions can lead to fibrosis [41], IFN-γ might induce corneal stromal fibrosis (or opacity) indirectly by increasing inflammation or directly by eliciting myofibroblast differentiation of corneal stromal fibroblasts. In keeping with this observation, our group previously demonstrated a correlation of corneal stromal opacity with levels of IFN-γ and IFN-γ^+^ CD4 cells in fucosyltransferase (FUT) 1 KO mice [42]. A similar observation was made by others in a study with HSV keratitis in which the amount of corneal scarring following HSV-1 reactivation was shown to correlate positively with higher levels of IFN-γ in both the cornea and trigeminal ganglia in mice [32]. Further studies will be required to elucidate the mechanisms through which IFN-γ induces myofibroblast differentiation in corneal stromal fibroblasts as well as how these functions may vary between different tissues.

Altogether, our data demonstrate a biological role of IFN-γ in induction of DED pathologies on the ocular surface at least in part through mechanisms that involve modulation of resident corneal cells, particularly corneal stromal fibroblasts, which are a principal cell population driving diseases of the cornea and ocular surface. Our results, together with the growing body of literature evaluating the association of IFN-γ and IFN-γ-producing cells with DED, suggest that targeting IFN-γ and corneal stromal fibroblasts may offer an effective strategy for treatment of DED, the most common ocular pathology.

## Materials and methods

### Cell culture

Primary human corneal epithelial cells and stromal fibroblasts were cultured from human donor corneas as previously reported [43, 44]. Donor corneas were obtained from Eversight (Ann Arbor, MI) in accordance with the provisions of the Declaration of Helsinki for research involving human tissue.

The epithelial cells were separated from the stroma by treatment with 10 mg/mL dispase II (Roche, Indianapolis, IN) for 1 h at 37 °C followed by 0.2 mg/mL EDTA (Lonza, Valais, Switzerland) for 5 min at 37 °C. The isolated cells were seeded on a mitomycin C (4 µg/mL, Sigma-Aldrich, St. Louis, MO)-pretreated 3T3 fibroblast feeder layer (NIH/3T3 cell line, ATCC, Manassas, VA) and cultured for 2–3 weeks under 5% CO_2_ at 37 °C in supplemented hormonal epithelial media containing DMEM/F12 (Welgene, Gyeongsan, Korea), 10% fetal bovine serum (FBS) (Gibco, Waltham, MA), 0.5% penicillin/streptomycin (Lonza), 5 µg/mL insulin, 30 ng/mL cholera toxin, 10 ng/mL epidermal growth factor, 0.18 mM adenine, 4 mM l-glutamine, and 2 nM triiodo-l-thyronine (all supplements from Sigma-Aldrich).

After removal of the epithelial cells, the corneal endothelium-Descemet membrane layer was peeled off from the cornea, and the remaining corneal stroma was treated with 1.2 U/mL dispase II (Roche Applied Science, Penzberg, Germany) and 200 U/mL type I collagenase (Worthington Biochemical Corp., Lakewood, NJ). The stromal cells were collected and cultured under 5% CO_2_ at 37 °C in DMEM/F12 (Welgene) containing 10% FBS (Welgene), 100 IU/mL penicillin/streptomycin (Lonza).

Passage 2 corneal epithelial and stromal cells were treated with 0–100 ng/mL recombinant human IFN-γ or IL-17A (R&D systems, Minneapolis, MN) for 18 h and subjected to assays.

### Cell viability assays

As an estimate of viable cell numbers, the metabolic activity of cells was quantified using Cell Counting Kit-8 (Dojindo Molecular Technologies, Rockville, MD), a colorimetric assay that measures the reduction of WST-8 tetrazolium salts to formazan by dehydrogenases in active mitochondria of living cells.

For determination of cytotoxicity, the activity of LDH released from cells into the cell culture medium was quantified using the CyQUANT LDH Cytotoxicity Assay (Abcam, Cambridge, UK) according to the manufacturer’s instructions.

### RT-qPCR

For RNA extraction, cells or tissues were lysed using RNA isolation reagent (RNA Bee, Tel-Test, Friendswood, TX) and homogenized with an ultrasound sonicator (Ultrasonic Processor, Cole Parmer Instruments, Vernon Hills, IL). Total RNA was isolated using the RNeasy Mini kit (Qiagen, Hilden, Germany) and converted to first-strand cDNA by reverse transcription (High Capacity RNA-to-cDNA Kit, Applied Biosystems, Carlsbad, CA). Then, real-time amplification was carried out using TaqMan Universal PCR Master Mix (Applied Biosystems) in the ABI 7500 Real-Time PCR System (Applied Biosystems). All PCR probe sets (TaqMan Gene Expression Assay kits) were purchased from Applied Biosystems. Data were normalized to *Gapdh* and expressed as fold changes relative to controls.

### Enzyme-linked immunosorbent assay (ELISA)

The cell-free supernatants were collected after centrifugation and assayed for concentrations of MCP-1/CCL2, IL-6, IL-8, and IL-1β using the DuoSet ELISA kit (R&D Systems, Minneapolis, MN).

### Flow cytometry

The cells were stained with fluorescence-conjugated antibodies against MHC class II, CD40, CD80, and CD86 (all from eBioscience, San Diego, CA) for 30 min at 4 °C. The stained cells were assayed for fluorescence using the S1000EXi Flow Cytometer (Stratedigm, San Jose, CA). The data were analyzed using the FlowJo program (BD, Franklin Lakes, NJ).

### Immunocytochemistry

For α-SMA immunostaining, cultured corneal stromal fibroblasts were fixed with 100% methanol for 5 min, permeabilized with 0.1% Triton X-100 for 5 min, and incubated in 1% bovine serum albumin blocking buffer for 1 h. Subsequently, the cells were incubated with mouse monoclonal anti-human α-SMA antibody (Cat No. ab7817, Abcam) overnight at 4 °C and then again with goat anti-mouse IgG secondary antibody (Cat No, A11001, Invitrogen, Waltham, MA) for 1 h at room temperature. A fluorescent mounting medium with DAPI (GBI, Rockville, MD) was used for nuclear counterstaining. The numbers of α-SMA-stained cells and total cells were counted in ×200 high-power fields (HPF), and the percentage of α-SMA-stained cells relative to the total number of cells was calculated.

### Animal model

The experimental protocols were approved by the Institutional Animal Care and Use Committee of Seoul National University Hospital and adhered to the ARVO Statement for the Use of Animals in Ophthalmic and Vision Research.

Eight-week-old male BALB/c mice were obtained from DooYeol Biotech (Seoul, Korea) and housed at a facility in the AAALAC-accredited Biomedical Research Institute of Seoul National University Hospital.

Under anesthesia by intramuscular injection of zolazepam-tiletamine (Zoletil, Virbac, Carros, France) and topical administration of 0.5% proparacaine ophthalmic solution (Hanmi Pharmaceutical, Seoul, Korea), recombinant mouse IFN-γ (10 or 100 μg/mL in 5 μL PBS, R&D Systems) was subconjunctivally injected near the superior limbus using a 30-gauge Neuros Syringe (Hamilton Company, Reno, NV) under an operating microscope (Carl Zeiss, Jena, Germany). The same volume of PBS (5 μL) was injected in the same way into the control group. Blinding and randomization were used during animal allocation, the conduct of experiments, the outcome assessment, and the data analysis.

### Clinical examination of corneal epithelial defects and stromal opacity

The corneas were clinically examined under slit-lamp biomicroscopy and photographed with a camera mounted on a surgical operating microscope.

Punctate epithelial erosions were observed after corneal vital staining with 3% (v/v) lissamine green dye (Sigma-Aldrich), and the extent of staining was graded using the standardized scale system (score 0: no staining; score 0.5: trace; score 1: less than one-third; score 2: less than two-thirds; score 3: more than two-thirds staining of the cornea) [45].

Corneal stromal opacity was evaluated based on the opacification scoring system (score 0: no opacity; score 1: slight superficial opacity, iris details visible; score 2: moderate stromal opacity, pupillary margin visible, iris details not visible; score 3: significant stromal opacity, pupillary margin not visible; score 4: completely opaque, pupil and anterior chamber not visible) [46].

Corneal epithelial defects and opacity were graded independently by two experienced ophthalmologists (S.K. and J.Y.O.) blinded to the treatment conditions.

### Phenol red thread test

The volume of aqueous tear production was quantified by phenol red thread test (Zone-Quick, Showa Yakuhin Kako, Tokyo, Japan). The folded end of a phenol red-impregnated cotton thread was hooked over the lateral one-third of the lower eyelid margin. After 15 s, the length of the tear-wetted thread was measured in mm.

### Histopathology

The tissues (the cornea, the conjunctiva including the superior and inferior forniceal conjunctiva, and the lacrimal glands including the exorbital lacrimal gland, intraorbital lacrimal gland, and Harderian gland) were excised and subjected to histologic or molecular assay.

For the histology, the tissues were fixed in 10% (v/v) formaldehyde, cut into 4-μm-thick sections, and subjected to hematoxylin and eosin staining. In addition, PAS staining (Cat No. ab150680, Abcam) was performed on the conjunctival sections. Immunohistochemistry for neutrophil and CD3 was performed in the corneal and lacrimal gland sections, respectively. For neutrophil immunofluorescence staining, rat monoclonal anti-mouse neutrophil antibody for murine Gr-1 (Cat No. ab2557, Abcam) was used as a primary antibody, and goat polyclonal anti-rat IgG antibody (Cat No, A10522, Invitrogen) as a secondary antibody. For CD3 immunostaining, rabbit polyclonal anti-CD3 antibody (Cat No. ab5690, Abcam) was used.

The number of PAS-stained cells in the conjunctiva N/Awas counted in four different sections through the superior and inferior fornices in each eye by two independent researchers (J.S.R. and H.J.S.), and the average number per section in each eye was determined as the GC count.

### Statistical analysis

All animals, experimental units, and data points were included in the analysis. Prism software (GraphPad, San Diego, CA) was used for statistical tests and graph generation. The D’Agostino & Pearson test or Shapiro–Wilk test was used for confirmation of normal distribution of data in each group. One-way ANOVA with Tukey’s test was applied for the comparison of the mean values from more than two groups. The Student’s *t* test was used for the comparison of values between the two groups. Data are presented as mean ± SD or SEM. Differences were considered significant at *P* < 0.05.

## Supplementary information

Supplementary Figures

## Data Availability

All data generated or analyzed during this study are included in this published article and its supplementary information files, and are available from the corresponding author on reasonable request.
