# Supplementary material for: Interferon-γ elicits the ocular surface pathology mimicking dry eye through direct modulation of resident corneal cells
Source: Cell Death Discov. 2023 Jun 30;9:209. doi: 10.1038/s41420-023-01511-0 (PMC10313777; doi:10.1038/s41420-023-01511-0)
Supplement: Supplementary file 1 — Supplementary Figures [file 41420_2023_1511_MOESM1_ESM.docx]

**Supplementary figures**

**
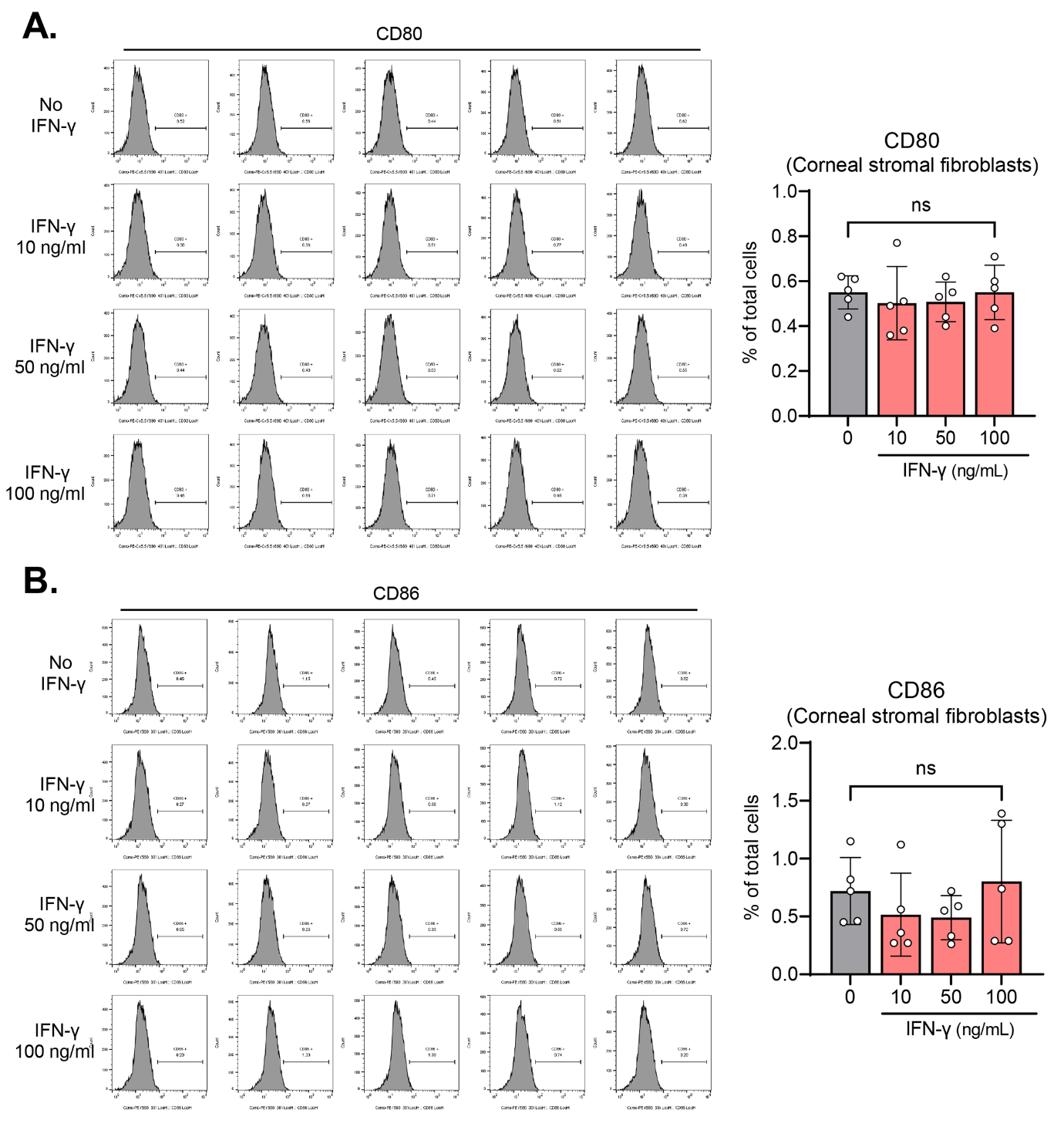
 Figure S1. IFN-γ does not affect the expression of CD80 or CD86 on corneal stromal fibroblasts.**

Representative flow cytometry histograms and quantitative results of CD80 (A) and CD86 (B) in human corneal stromal fibroblasts treated with IFN-γ or IL-17. Mean values ± SD are presented. ns: not significant as analyzed by one-way ANOVA

**
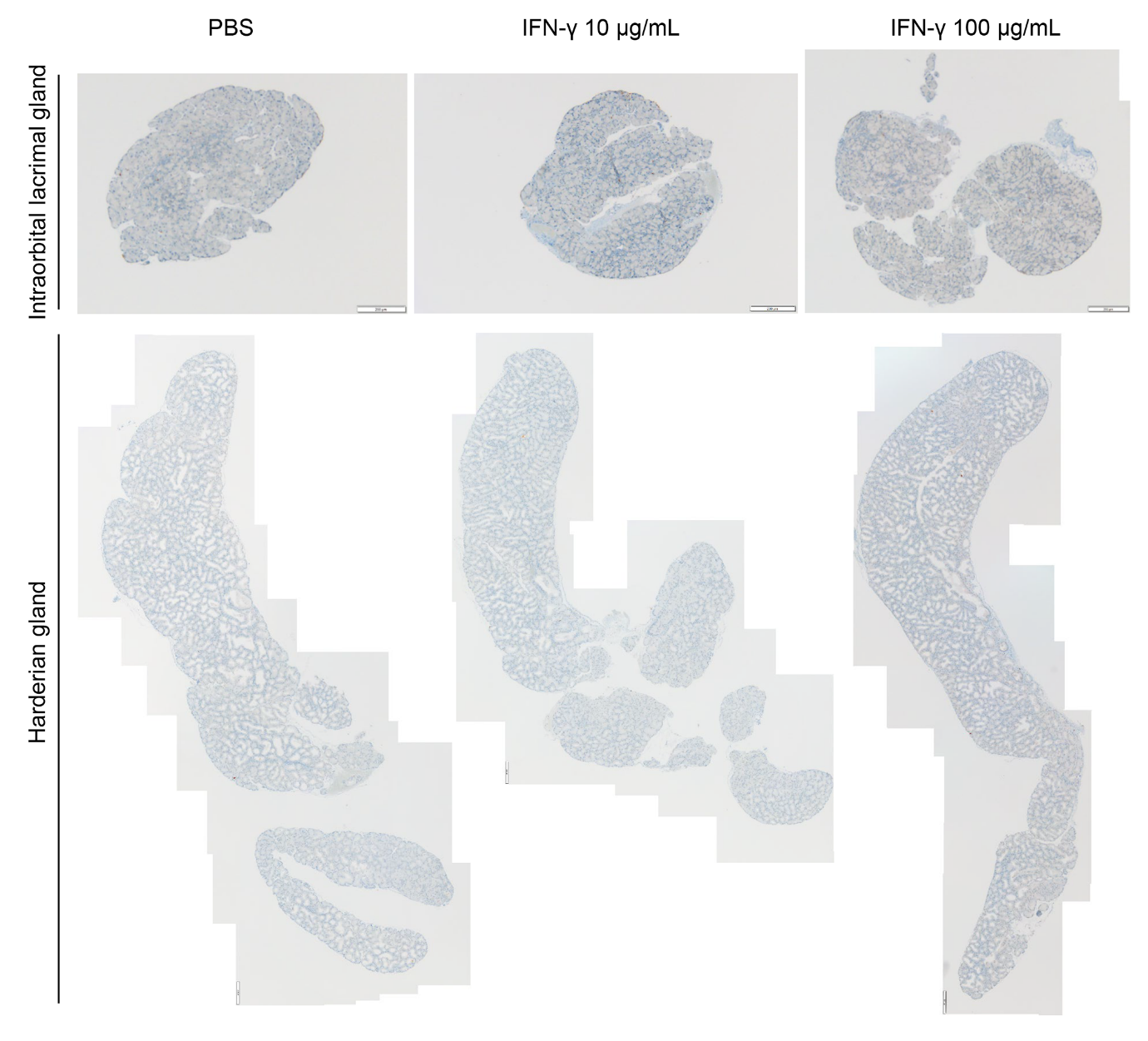
**

**Figure S2. IFN-γ does not induce T cell infiltration into the intraorbital lacrimal gland or Harderian gland.**

Representative microphotographs of CD3 immunostaining of intraorbital lacrimal gland and Harderian gland in mice 7 days after subconjunctival IFN-γ injection (10 or 100 μg/mL). Magnification ×100. Scale bar: 200 μm
